# Core *cis*‐element variation confers subgenome‐biased expression of a transcription factor that functions in cotton fiber elongation

**DOI:** 10.1111/nph.15063

**Published:** 2018-02-21

**Authors:** Bo Zhao, Jun‐Feng Cao, Guan‐Jing Hu, Zhi‐Wen Chen, Lu‐Yao Wang, Xiao‐Xia Shangguan, Ling‐Jian Wang, Ying‐Bo Mao, Tian‐Zhen Zhang, Jonathan F. Wendel, Xiao‐Ya Chen

**Affiliations:** ^1^ National Key Laboratory of Plant Molecular Genetics National Center for Plant Gene Research Institute of Plant Physiology and Ecology/CAS Center for Excellence in Molecular Plant Sciences University of CAS Chinese Academy of Sciences Shanghai 200032 China; ^2^ Plant Stress Biology Center Institute of Plant Physiology and Ecology/CAS Center for Excellence in Molecular Plant Sciences University of CAS Chinese Academy of Sciences Shanghai 200032 China; ^3^ Plant Science Research Center Shanghai Key Laboratory of Plant Functional Genomics and Resources Shanghai Chenshan Botanical Garden Shanghai 201602 China; ^4^ Department of Ecology, Evolution and Organismal Biology Iowa State University Ames IA 50011 USA; ^5^ Nanjing Agricultural University Nanjing Jiangsu 210095 China; ^6^ Zhejiang University Hangzhou Zhejiang 310058 China

**Keywords:** allopolyploid, *Gossypium hirsutum*, Homoeolog, molecular evolution, *PRE1*, TATA‐box

## Abstract

- Cotton cultivars have evolved to produce extensive, long, seed‐born fibers important for the textile industry, but we know little about the molecular mechanism underlying spinnable fiber formation. Here, we report how *PACLOBUTRAZOL RESISTANCE 1* (*PRE1*) in cotton, which encodes a basic helix‐loop‐helix (bHLH) transcription factor, is a target gene of spinnable fiber evolution.
- Differential expression of homoeologous genes in polyploids is thought to be important to plant adaptation and novel phenotypes. *PRE1* expression is specific to cotton fiber cells, upregulated during their rapid elongation stage and A‐homoeologous biased in allotetraploid cultivars. Transgenic studies demonstrated that PRE1 is a positive regulator of fiber elongation.
- We determined that the natural variation of the canonical TATA‐box, a regulatory element commonly found in many eukaryotic core promoters, is necessary for subgenome‐biased *PRE1* expression, representing a mechanism underlying the selection of homoeologous genes.
- Thus, variations in the promoter of the cell elongation regulator gene *PRE1* have contributed to spinnable fiber formation in cotton. Overexpression of *GhPRE1* in transgenic cotton yields longer fibers with improved quality parameters, indicating that this bHLH gene is useful for improving cotton fiber quality.

## Introduction

Polyploids, which harbor two or more sets of genomes in their nuclei, are common in flowering plants (Soltis *et al*., 2014). Many crops are neo‐allopolyploids, such as the upland cotton *Gossypium hirsutum* and the extra‐long staple (ELS) cotton *G. barbadense*, both of which are allotetraploids (Gong *et al*., 2013; Renny‐Byfield & Wendel, 2014; Wendel & Grover, 2015). Exploring the mechanisms that regulate the expression of homoeologous gene pairs may generate clues regarding the origin of important traits that have arisen during the evolution and domestication of polyploid crops (Fang *et al*., 2017b; Wang *et al*., 2017).

Cotton is the major source of natural fiber for the textile industry. Cotton fibers, which are single‐celled, epidermal seed trichomes, have two remarkable features: extreme length (may exceed 4 cm) and high (> 95%) cellulose content at maturity (Kim & Triplett, 2001; Mansoor & Paterson, 2012). After initiation from the outer integument before the day of anthesis, cotton fiber cells undergo rapid primary cell wall elongation until *c*. 2–3 wk after anthesis, before entering into secondary cell wall synthesis (Applequist *et al*., 2001; Lee *et al*., 2007; Mansoor & Paterson, 2012).

A series of transcriptional factors involved in regulation of cotton fiber development have been reported. Both transgenic and genetic mapping results indicate that the MIXTA‐type R2R3 MYB (myeloblastosis) transcription factor MYB25‐like and its homoeologs function as the master regulator of fiber cell initiation and early development (Walford *et al*., 2011; Tan *et al*., 2016; Wan *et al*., 2016; Wu *et al*., 2018). Additionally, the GL1‐type MYBs may also play a role, because all homoeologs of the *Arabidopsis* MYB‐bHLH‐WD40 (MBW) complex (Szymanski *et al*., 2000) are expressed in cotton fibers (Shangguan *et al*., 2008). During elongation, the phytohormone gibberellin (GA) plays a key role in promoting fiber growth, during which GhHOX3, an homoeodomain leucine zipper (HD‐ZIP) IV transcription factor, acts as a core regulator (Rombola‐Caldentey *et al*., 2014; Shan *et al*., 2014). Because cell elongation involves multiple signaling pathways and cotton fiber cells are unique in their extensive and synchronous elongation, isolation of new regulators will further our understanding of the molecular basis of cotton fiber development and of plant cell growth in general.

The cotton genus *Gossypium* (Malvaceae) includes > 50 species (Wendel & Grover, 2015). None of the *c*. 45 diploids (2*n* = 26), which are divided into eight (A to G and K) genome groups (Wendel & Grover, 2015), produce spinnable fiber except for the two A‐genome species *G. herbaceum* and *G. arboreum*. Allopolyploid species formed *c*. 1–2 Myr ago through hybridization between A and D genome species (Wendel *et al*., 2010; Wendel & Grover, 2015), and accordingly, they contain two subgenomes, designated At and Dt (with the t denoting subgenome in the tetraploid). Of the seven allotetraploid cotton species identified to date (Grover *et al*., 2014, 2015; Gallagher *et al*., 2017), *G. hirsutum* and *G. barbadense* have been domesticated and are now widely cultivated for fiber and other byproducts (Wendel *et al*., 2009, 2010; Renny‐Byfield *et al*., 2016).

Recent progress in genomic analyses of cottons (Paterson *et al*., 2012; Wang *et al*., 2012; Li *et al*., 2014, 2015; Liu *et al*., 2015; Zhang *et al*., 2015) have generated extensive data for cotton research and breeding. The tetraploid cottons have a relatively large genome of *c*. 2.5 Gb, > 60% comprising repetitive sequences (Li *et al*., 2015; Zhang *et al*., 2015). The two subgenomes, At and Dt, are largely collinear and syntenic, although they differ approximately two‐fold in genome size (Paterson *et al*., 2012; Wang *et al*., 2012; Li *et al*., 2014, 2015; Liu *et al*., 2015; Wendel & Grover, 2015; Zhang *et al*., 2015). Polyploidization in plants is known to induce a wide spectrum of genomic changes and novel regulatory interactions (Wendel, 2015), at least some of which are likely to facilitate phenotypic innovation and be relevant to cotton evolution and cotton fiber improvement (Otto, 2007; Wendel *et al*., 2010; Renny‐Byfield *et al*., 2016). Transcriptomic analyses have demonstrated that in both *G. hirsutum* and *G. barbadense*, > 20% of genes show subgenome‐biased expression in specific tissues (Yoo & Wendel, 2014; Liu *et al*., 2015; Zhang *et al*., 2015), but little is understood regarding the molecular basis of differential homoeologous gene expression.

In the present study, we characterize a basic Helix‐Loop‐Helix (bHLH) factor, PRE1 (PACLOBUTRAZOL RESISTANCE 1) from *G. hirsutum* (*Gh*), which we observed to be preferentially expressed in fiber cells. The bHLH proteins constitute a large family of transcription factors in eukaryotes (Zhiponova *et al*., 2014). Typically, a bHLH domain harbors two functional regions, the N‐terminal basic region responsible for DNA binding, and the C‐terminal HLH region which mediates the formation of either homo‐ or heterodimers of bHLH proteins (Wang *et al*., 2009; Zhang *et al*., 2009; Oh *et al*., 2014). In addition, some members of the family are atypical in lacking the DNA‐binding basic region; these are thought to mediate the activities of other transcription factors through protein–protein interactions (Wang *et al*., 2009). Among atypical bHLHs, members of the inhibitor of DNA binding/differentiation (Id) in animals (Ling *et al*., 2014) and the paclobutrazol resistance (PRE) family in plants are well‐characterized (Bai *et al*., 2012; Zhiponova *et al*., 2014). The PREs are mostly small proteins composed of 90–110 amino acid residues, with highly conserved HLH domains but other, more variable regions. The *Arabidopsis thaliana* PRE1 was first identified from a mutant insensitive to paclobutrazol (PAC), a GA biosynthesis inhibitor, and promotes elongation of hypocotyl cells (Lee *et al*., 2006). Another PRE of *Arabidopsis*, PRE6, was also reported to stimulate hypocotyl growth (Hyun & Lee, 2006).

Here, we assess the function of *GhPRE1* in promoting fiber elongation and describe the evolution of the *PRE1* promoter and its possible relationship to the long‐fiber phenotype. We show that in fibers of most of the allopolyploid cotton species, only the A‐subgenome copy is expressed, which provides evidence that variation in the TATA‐box region is the primary factor behind the differential homoeologous *PRE1* expression in allotetraploid cottons. This is the first demonstration of a molecular mechanism that underlies differential homoeolog expression in cotton, potentially connecting homoeolog bias to an important plant phenotype.

## Materials and Methods

### Plant materials and growth conditions

The authorities for all of the cotton species under our investigation include: the Institute of Plant Physiology and Ecology, Chinese Academy of Sciences, China; the National Wild Cotton Nursery, China; Nanjing Agriculture University, China; and Iowa State University, USA.

Cotton species used include the allopolyploid species *Gossypium hirsutum* (*Gh;* 33 accessions of *G. hirsutum* were used; see Supporting Information Table S1); *G. barbadense*;* G. tomentosum*;* G. mustelinum*;* G. darwinii*;* G. ekmanianum*; the two A‐genome diploids *G. herbaceum* and *G. arboretum*; the D‐genome species closest to the Dt genome donor, *G. raimondii*; and 17 other diploid species from across the genus (with one outgroup, *Thespesia populnea*), as listed in Table S1. These wild diploid and allopolyploid *Gossypium* materials were obtained from the National Wild Cotton Nursery of Cotton Research Institute, Chinese Academy of Agricultural Sciences, Sanya, Hainan, China.

**Figure 1:**
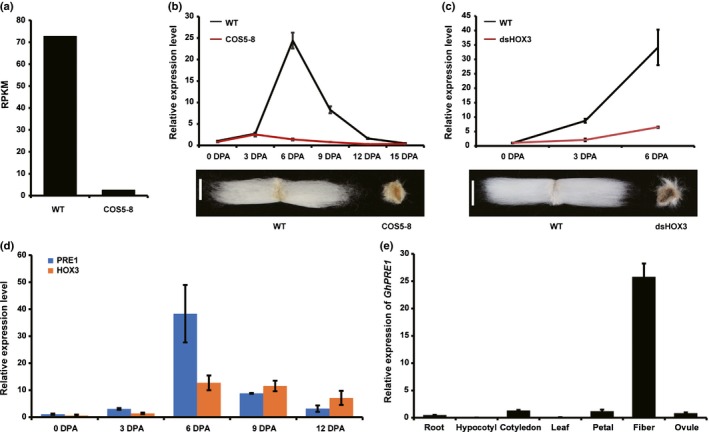
*Gossypium hirsutum PACLOBUTRAZOL RESISTANCE 1* (*GhPRE1*) is highly expressed in cotton fiber cells during elongation. (a) *GhPRE1* transcript abundance in the transcriptome of the 6‐d post‐anthesis (DPA) cotton fiber of *Gossypium hirsutum*, which was greatly reduced in the *35S::GhHOX3* co‐suppression line (*COS5‐8*) compared to wild‐type (WT). RPKM, reads per kilobase per million mapped reads. (b, c) Expression of *GhPRE1* in cotton fiber cells during elongation, which was nearly completely repressed in (b) *COS5‐8* and (c) *35S::dsGhHOX3*; bars, 1 cm. (d) Expressions of *GhPRE1* and *GhHOX3* in cotton fiber cells, which show similar dynamic patterns during fiber elongation and a peak at *c*. 6 DPA. (e) Expression of *GhPRE1* in different cotton tissues, showing a high specificity to cotton fiber. From (b–e), the relative transcript levels were analyzed by quantitative reverse transcription PCR (qRT‐PCR), with cotton *histone‐3* (AF024716) as the internal reference. Error bars indicate ± SD (*n *=* *3).

Plants of *G*.* hirsutum* cv R15 and its transgenic lines were grown in the glasshouse at 28 ± 2°C under a 14 h : 10 h, light : dark photoperiod, and in the field in Songjiang (Shanghai), Anyang (Henan Province) and Sanya (Hainan Province). *Gossypium barbadense* cv Xinhai 21*, G. darwinii, G. arboreum* and *G. herbaceum* were also cultivated. The rest of the species were collected from the cotton nursery in Sanya and Nanjing Agriculture University, China, or from Iowa State University, USA. Ovules were harvested at different growth stages as indicated in Figs 1 and (see later) 3 and 5, and fibers were isolated by scraping the ovule in liquid nitrogen. Plants of *Nicotiana benthamiana* and *Arabidopsis thaliana* (Col‐0) were grown at 28 ± 2°C or 22 ± 2°C, respectively, under a 16 h : 8 h, light : dark photoperiod.

### Plant transformation and phenotypic analysis

The open reading frame (ORF) of *PACLOBUTRAZOL RESISTANCE 1* (*GhPRE1*) was PCR‐amplified from a *G. hirsutum* cv R15 fiber cDNA library with PrimeSTAR HS DNA polymerase (Takara Biomedical Technology Co. Ltd, Beijing, China) and inserted into the *pCAMBIA 2301* vector to construct *35S::GhPRE1A* and *RDL1::GhPRE1A*. For *35S::dsPRE1*, sense and antisense *GhPRE1A* fragments, separated by a 120‐bp intron of the *RTM1* gene from *A. thaliana*, were cloned into *pCAMBIA2301*. Primers used in this investigation are listed in Table S2.

**Figure 2:**
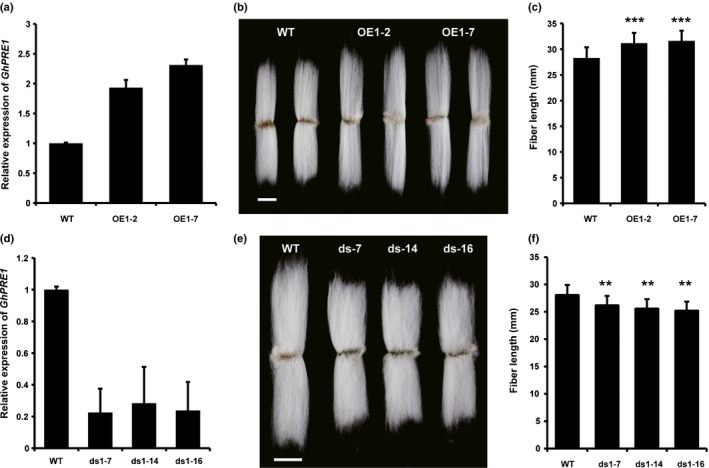
*Gossypium hirsutum PACLOBUTRAZOL RESISTANCE 1* (*GhPRE1*) promotes cotton fiber elongation. (a–c) Expression of *GhPRE1* in 6‐d post‐anthesis (DPA) fibers in (a) the *RDL1::GhPRE1* overexpression (OE) lines, their fiber (b) phenotypes and (c) length compared to wild‐type (WT). (d) Expression of *GhPRE1* in three RNAi (*35S::dsGhPRE1*) lines, (e) their fiber phenotypes and (f) length. Expression was analyzed by quantitative reverse transcription PCR (qRT‐PCR); error bars indicate SD (*n *=* *3). Bars, 1 cm. Fiber length data were analyzed by Student's *t*‐test compared to WT: **, *P *≤* *0.01; ***, *P *≤* *0.001). Error bars represent SD. (*n *=* *30).

The binary constructs were transferred into *Agrobacterium tumefaciens*. Plants of *A. thaliana* were transformed with a flower dip method (Bent, 2006). Cotton transformation was performed as described previously (Shangguan *et al*., 2008). Briefly, hypocotyl segments of the 5‐ to 7‐d‐old cultivated *G. hirsutum* cv R15 seedlings were used as explants for *A. tumefaciens* infection*,* calluses were induced and proliferated, and plantlets were then regenerated. Transgenic cotton plants were grown in glasshouse or field. For T_0_ and subsequent generations, β‐glucuronidase (GUS) histochemistry staining and PCR were carried out to identify the transgenic lines. To measure fiber length, 30 seeds each plant were harvested at random, fibers were swept to two sides by a comb and length was measured with a ruler (Figs 1, 2).

### Fiber quality measurements

The fiber quality parameters, including fiber length, fiber strength, micronaire and spinning consistency, were measured at the Cotton Fiber Quality Inspection and Test Center of the Ministry of Agriculture (Anyang, China) with an HVI 900 instrument (Uster Technologies, Shanghai, China).

### Nucleic acid isolation and gene expression analysis

Genomic DNA of different cotton species was isolated using a cetyltrimethyl ammonium bromide (CTAB) extraction solution (2% CTAB, 0.1MTris, 20 mM EDTA, 1.4M NaCl, pH = 9.5), as described (Stewart & Via, 1993). For RNA extraction, the samples were ground in liquid nitrogen and total RNAs were extracted using the RNAprep Pure Plant Kit (Tiangen Biotech Co. Ltd, Beijing, China). The coding sequence of cotton *PRE1* (*GhPRE1A*) was cloned by 5′‐ and 3′‐RACE according to the manufacturer's instructions (TaKaRa). Total RNAs of 1 μg were used for cDNA synthesis with oligo (dT) primers and M‐MLV (Moloney Murine Leukemia Virus) Reverse Transcriptase (Invitrogen). The products were diluted 10‐fold before analysis. Quantitative real‐time PCR was performed with SYBR‐Green PCR Mastermix (TaKaRa), and amplification was real‐time monitored on a cycler (Mastercycler RealPlex; Eppendorf Ltd, Shanghai, China). Cotton *histone‐3* gene (*GhHIS3*, AF024716) was used as the internal reference. Transcriptome analysis of expressions is based on the previously generated data (Shan *et al*., 2014; Yoo & Wendel, 2014; Zhang *et al*., 2015).

### Microscope observation

A *35S::GhPRE1A‐VENUS* in *pCAMBIA2301* vector was constructed to determine subcellular localization of the protein. *Agrobacterium* cells containing the plasmid were infiltrated into the abaxial side of *N. benthamiana* leaves by using a syringe for transient expression. The infiltrated areas were harvested 72 h later and observed under a confocal microscope (LSM510, Zeiss). Hypocotyl epidermal cells of *A. thaliana* were wet‐mounted and observed under an optical microscope.

### Sequence polymorphism analysis

The genomic sequence of *PRE1* was first cloned from *G. hirsutum* cv R15 by using the Genome Walking kit (TaKaRa). *PRE1* locus was re‐examined by local blast with the reported genome sequence data (Wang *et al*., 2012; Li *et al*., 2014; Liu *et al*., 2015; Zhang *et al*., 2015) using the software BioEdit (http://www.mbio.ncsu.edu/bioedit/bioedit.html). The genomic sequence of *PRE1* and a 3‐kb upstream sequence flanking to this locus (Table S3) were picked for further analysis. For diploid and allotetraploid cotton species whose genome sequences were unavailable, the *PRE1* gene fragment was obtained by PCR, followed by insertion into the PMD18‐T vector for sequencing. The sequences in different Upland cotton (*G. hirsutum*) or extra‐long staple (ELS) cotton (*G. barbadense*) cultivars were obtained from the reported data (Fang *et al*., 2017a).

The software Vector NTI was used to align *PRE1* sequences. Homoeologous comparisons were conducted between A and D subgenomes in polyploids. Mega 7 software (http://megasoftware.net/) was used to construct phylogenetic trees with maximum‐likelihood estimation and 1000 bootstrap resamplings.

### Pyrosequencing

*GhPRE1A* was amplified from the fiber cDNA library of *G. hirsutum* cv R15 and inserted into the pMD18‐T vector. For *GhPRE1D*, the predicted cDNA was synthesized and inserted into the pUC57 vector (Sangon Biotech Co. Ltd, Shanghai, China). Artificial mixtures were generated by adding the PCR products of the two genes in a series of defined ratios as shown in Fig. S1(a), and subjected to pyrosequencing (Schaart *et al*., 2005; Wang & Elbein, 2007) (Sangon Biotech). For expression analysis *in vivo*, cDNAs were generated by reverse transcription of total RNAs with oligo (dT) primers and subject to pyrosequencing. The relative expression levels of *PRE1* were determined by using primers list in Table S2.

### Promoter activity analysis

The 0.7‐kb promoter fragments of *PRE1* were amplified from *G. hirsutum* cv R15 genomic DNA and inserted into the luciferase reporter. The deletion of the TATA‐box from the *GhPRE1A* promoter and its insertion in the *GhPRE1D* promoter were performed by overlapping PCR with the primers listed in Table S2. The plasmids were transferred into *A. tumefaciens*, together with a co‐suppression repressor plasmid, pSoup‐P19. The transformed cells were collected and were infiltrated into the abaxial side of *N. benthamiana* leaves with a syringe. The infected areas were harvested 3 d later for extraction of total proteins, which were analyzed by using the kit Dual‐Luciferase Reporter Assay System (Promega; E1910). The fluorescent values of firefly luciferase (LUC) and Renilla (REN) luciferase were detected with Promega GloMax 20/20, according to the manufacturer's instructions. The value of LUC was normalized to that of REN. Promoter activities in cotton fiber cells were determined by transient expression of a green fluorescence protein reporter. The 0.7‐kb promoter fragments of *GhPRE1* were fused to the coding region of a green fluorescent protein (GFP) reporter in *pCAMBIA2301* (Fig. S2a). The plasmids were transformed into ovules (1DPA) by helium‐driven particle accelerator (PDS‐1000; Bio‐Rad). The ovules were incubated for 24 h at 30°C in the dark before observation of GFP signal in fiber cells under a confocal laser scanning microscopy (LSM510; Zeiss).

## Results

### *PRE1* promotes cotton fiber elongation

The homeodomain leucine zipper (HD‐ZIP) IV transcription factor GhHOX3 is a key regulator of cotton fiber elongation, as demonstrated by the fact that both *35::GhHOX3* transgene co‐suppression (*COS5‐8*) and *35S::dsGhHOX3* RNA interference (RNAi) lines of *G. hirsutum* produce shorter, fuzzier fibers (Shan *et al*., 2014). Transcriptome comparisons revealed that one of the PRE family genes, *GhPRE1*, was markedly downregulated in *COS5‐8* cotton fiber (Fig. 1a). Analysis by real‐time quantitative reverse transcription PCR (qRT‐PCR) confirmed its downregulation following *GhHOX3* silencing and indicated its high expression in fiber cells during 6–9 d post‐anthesis (DPA) when the fiber underwent rapid elongation (Fig. 1b,c). The similar expression pattern shared with *GhHOX3* (Fig. 1d) and the highly specific expression in fiber cell (Fig. 1e) suggest that PRE1 may function in modulating fiber cell elongation.

Transient expression assays with tobacco leaf cells localized the fusion protein of GhPRE1A‐VENUS (the letter after the gene name denotes genome or subgenome) to the nucleus and the plasma membrane (Fig. S3a). When introduced into *A. thaliana*, seedlings overexpressing *GhPRE1A* produced longer hypocotyls and roots (Fig. S3b–e), demonstrating that cotton PRE1 functions in a similar way to *Arabidopsis* homoeologs in promoting cell elongation (Lee *et al*., 2006).

In order to provide direct evidence of *GhPRE1* function in cotton, we engineered *G. hirsutum* for overexpression and suppression. The promoter of cotton *RDL1* gene is strong and fiber/trichome‐specific (Wang *et al*., 2004; Xu *et al*., 2013). When *GhPRE1A* was expressed in transgenic *G. hirsutum* under the control of the *RDL1* promoter, the transcript levels increased in fiber cells (Fig. 2a) and the *RDL1::GhPRE1A* lines produced longer fibers compared to the untransformed control (Fig. 2b,c). Apart from mature fiber length, other fiber quality parameters of the overexpression lines, such as strength and micronaire, were also improved (Table 1). On the contrary, suppressing *GhPRE1* expression by RNAi resulted in shorter fibers (Fig. 2d,e,f). Although the difference was notable and significant, the fiber length reduction was not as severe as that caused by *GhHOX3* silencing (Shan *et al*., 2014), probably due to insufficient knockdown and functional redundancy of *PRE* family members. Together, these data indicate that *GhPRE1* is highly and preferentially expressed in cotton fiber cells where it acts as a positive regulator of cell elongation.

**Table 1:** Fiber quality parameters of the wild‐type (WT) and the *Gossypium hirsutum PACLOBUTRAZOL RESISTANCE 1* (*GhPRE1*) overexpression (OE) cotton lines

Genotype	Len	Str	Mic	SCI
WT	30.05 ± 0.35	28.6 ± 0.98	4.85 ± 0.07	147.50 ± 0.71
OE1‐2	32.55 ± 0.63	29.75 ± 0.35	4.40 ± 0.28	154.00 ± 2.82
OE1‐7	32.75 ± 0.49	31.25 ± 0.07	4.40 ± 0.01	162.00 ± 7.07

Len, length; Str, strength; Mic, micronaire; SCI, spinning consistency. Error values indicate ± SD (*n *=* *3).

### Expression of *PRE1* in cottons is related to long fiber formation

**Figure 3:**
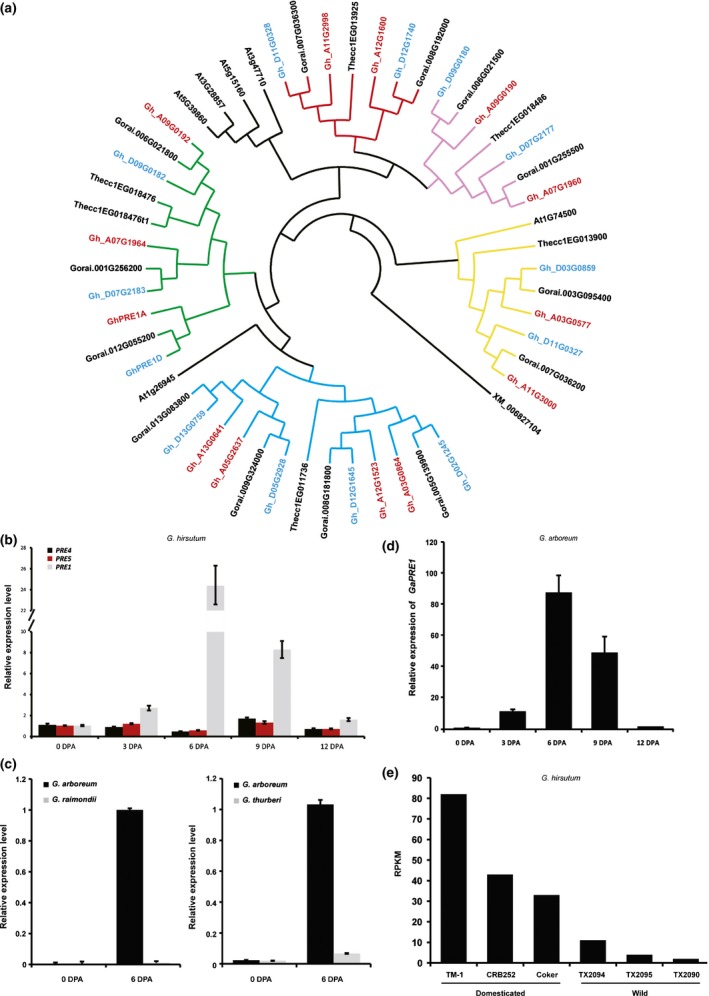
*PACLOBUTRAZOL RESISTANCE 1* (*PRE1*) expression associates with spinnable cotton fiber formation. (a) Phylogenetic analysis of *PRE* genes in *Theobroma cacao* (Thecc), *Arabidopsis thaliana* (At), *Gossypium raimondii* (Gorai) and *G. hirsutum* (Gh) by the maximum‐likelihood on nucleotide sequences. Subfamilies are denoted by different colors, with *PRE1* being in the red clade. *GhPRE1A/D*, Gh_A05G3166_5/Gh_D04G0454; *GhPRE2A/D*, Gh_A12G1523/Gh_D12G1645; *PRE3A/D*, Gh_A03G0864/Gh_D02G1245; *PRE4A/D*, Gh_A07G1964/Gh_D07G2183; *PRE5A/D*, Gh _A09G0192/Gh_D09G0182; *PRE6A/D*, Gh_A03G0577/Gh_D03G0859; *PRE7A/D*, Gh_A05G2637/Gh_D05G2928; *PRE8A/D*, Gh_A07G1960/Gh_D07G2177; *PRE9A/D*, Gh_A09G0190/Gh_D09G0180; *PRE10A/D*, Gh_A11G3000/Gh_D11G0327; *PRE11A/D*, Gh_A11G2998/Gh_D11G0328; *PRE12A/D*, Gh_D12G1740/Gh_A12G1600; *PRE13A/D*, Gh_A13G0641/>Gh_D13G0759. The *PRE* ortholog from *Amborella trichopoda* (XM_006827104) was used as outgroup. (b) Expression of *GhPRE* genes in cotton fibers at different numbers of days post‐anthesis (DPA), as indicated, *GhPRE1* is the major *PRE* gene expressed in cotton fiber. (c) Expression of *PRE1* orthologs in fibers of the A‐genome species *G. arboreum* compared to two D‐genome species: left, *G. raimondii*, the model progenitor diploid to allopolyploid cottons, and right, *G. thurberi*, a phylogenetically more distant D‐genome species. (d) Expression of *PRE1* orthologs in the A‐genome *G. arboreum* fibers during the fast elongation stage. The transcripts were analyzed by quantitative reverse transcription PCR (qRT‐PCR); error bars indicate SD (*n *=* *3). (e) Expression levels of *GhPRE1* in domesticated *G. hirsutum* cultivars and wild relatives, based on transcriptome data of 10‐DPA fiber. RPKM, reads per kilobase per million mapped reads.

Changes in seed‐born trichome length are characteristic of cotton species. There are four cotton species domesticated for spinnable fiber, among which two (*G. hirsutum* and *G. barbadense*) are AtDt allotetraploids and two (*G. herbaceum* and *G. arboreum*) are A‐genome diploids. Although *G. hirsutum* generally is grown for its high fiber yield, *G. barbadense* produces ELS fibers for upmarket textiles. Our previous BLAST search with *Arabidopsis* PRE queries identified 13 *PRE* genes in *G. raimondii*, the best extant model of the D‐genome progenitor, and recovered all 26 predicted orthologs – 13 in each subgenome – in *G. barbadense* (Liu *et al*., 2015). The *G. hirsutum* genome also harbors the same expected 26 *PRE* genes (Fig. 3a). Although transcripts of several *PRE* genes were detected in *G. hirsutum* fiber, *GhPRE1* was predominant with regard to their mRNA levels (Figs 3b, S4a), which further suggests that *GhPRE1* is specialized to function in regulating cotton fiber elongation.

We then extended the expression analysis of *PRE1* to other cotton species. Analysis by qRT‐PCR showed that *PRE1* expression was undetectable in seeds of *G. raimondii*, or in other diploid species that do not bear long fibers, such as *G. thurberi* (Fig. 3c). However, the A‐genome *G. arboreum* (Fig. 3c,d), as well as three other fiber‐bearing allotetraploid cottons (*G. barbadense*,* G. darwinii* and *G. tomentosum*), all showed a clear expression of *PRE1* in fiber cells at the mid‐elongation stage (Figs S4b–d, 3). These data suggest that *PRE1* has probably acquired its expression in cotton seed trichomes along with long fiber evolution.

Furthermore, the correlation between *PRE1* expression and cotton fiber elongation is not limited to the interspecies level, because domesticated cultivars of *G. hirsutum* exhibit higher transcript levels than their respective non‐domesticated races (Fig. 3e), suggesting that the *PRE1* expression levels in fiber cells have been under selection during the course of domestication and crop improvement for longer fiber.

### *PRE1* sequence variation

**Figure 4:**
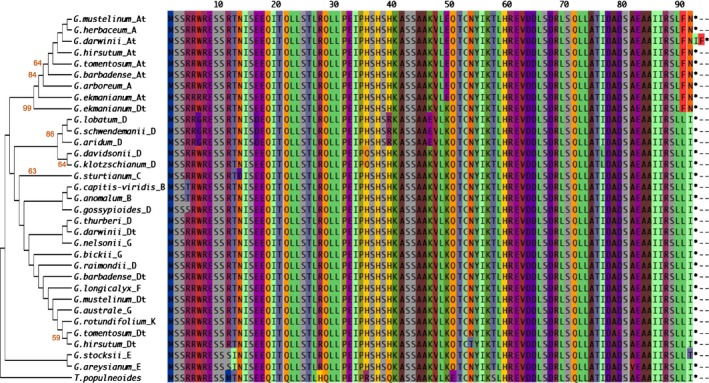
Phylogenetic tree and protein sequences of cotton *PACLOBUTRAZOL RESISTANCE 1* (*PRE1*). The phylogenetic relationship of 32 cotton PRE1 protein sequences (from 20 diploid and six allotetraploid species) was inferred by using MEGA and the maximum‐likelihood method based on the Jones–Taylor–Thornton (JTT) model with 1000 bootstrap replications. Percentage bootstrap scores of > 50% were displayed. Notice the putative gene conversion at positions 90 and 91 in the At homoeolog of *Gossypium ekmanianum*. The letter(s) after the species name denotes the genome or subgenome group, *Thespesia populnea* PRE1 was used as outgroup.

**Figure 5:**
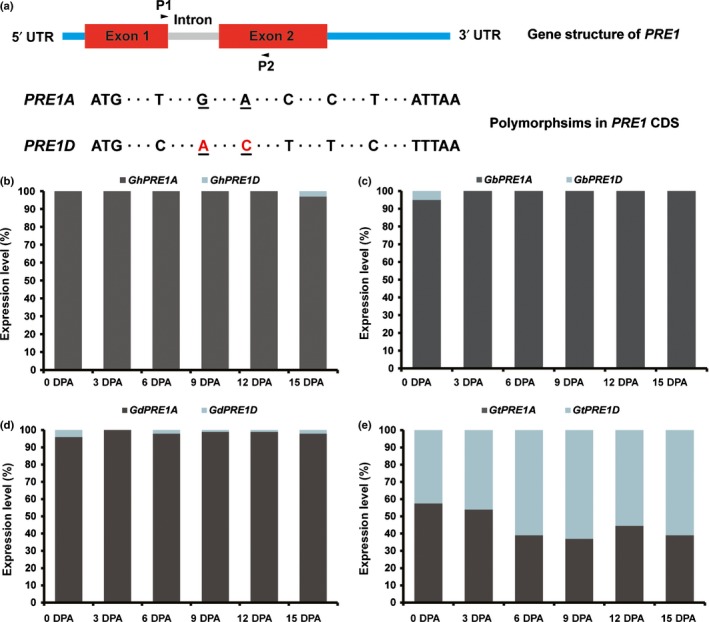
Subgenome‐biased expression of *PACLOBUTRAZOL RESISTANCE 1* (*PRE1*) in allotetraploid cottons. (a) Schematic representation of *PRE1* gene and nucleotide polymorphisms in the coding region of *PRE1* homoeologs (*PRE1A* and *PRE1D*) in allotetraploid cottons. P1 and P2 indicate locations of primers used in pyrosequencing. The single nucleotide polymorphisms (SNPs) used for genotyping the homoeologous pair are indicated by underlining. Identical sequences are not shown. (b–d) *PRE1* shows a striking expression bias toward the At homoeolog in ovules and fibers of (b) *Gossypium hirsutum*, (c) *G. barbadense* and (d) *G. darwinii*. (e) Both At and Dt copies of *PRE1* were expressed in ovules and fiber cells of *G. tomentosum*, a wild allotetraploid cotton species endemic to the Hawaiian Islands. All pyrosequencing assays were repeated three times with similar results, and representative results are shown. The routine technical error of pyrosequencing is *c*. 5%. DPA, days post‐anthesis.

We aligned homoeologous regions of *PRE1* coding sequence and its flanking regions from all diploid and allopolyploid species surveyed (Table S1). Gene structure was highly conserved among all species, with two exons of 40 and 51 amino acid residues, separated by an intron of 70 (*PRE1D* from *G. hirsutum* and *G. ekmanianum*) or 74 (all other species) nucleotides (Figs 4, 5a). *PRE1* nucleotide sequences also are highly conserved in the genus, with 13 and 16 polymorphic sites in Exons 1 and 2, respectively, corresponding to an average of three amino acid differences between each pair of sequences, and a maximum difference of eight amino acids between members of the D and E genome (Figs 4, 5a). Not surprisingly, phylogenetic analysis of the protein sequences led to trees that were incompletely resolved due to strong sequence conservation, yet sequences from related species generally fell into the same clades (Fig. 4). Interestingly, the alignments suggest historical evidence for interhomoeolog gene conversion, as has been reported for other cotton genomic regions (Salmon *et al*., 2010; Wang & Paterson, 2011). Specifically, the Dt homoeolog from the 3′ end of exon 2 in *G. ekmanianum* displayed a conversion track such that its inferred amino acid sequence contained two sequential residues that are otherwise restricted to the At homoeologs of all other allopolyploid species (Fig. 4).

### Subgenome bias of *PRE1* expression in allotetraploid cottons

Homoeologous genes may express unequally in a tissue, a phenomenon called expression bias (Yoo *et al*., 2013). Considering that allotetraploid cottons originated from hybridization between the A‐ and D‐genome progenitors *c*. 1–2 Myr ago (Wendel, 1989; Small *et al*., 1998; Wendel & Cronn, 2003; Paterson *et al*., 2012; Wendel & Grover, 2015), we asked whether the At and Dt homoeologs of *PRE1* are expressed equally. We further addressed whether expression in allotetraploid cotton fiber has been altered by polyploidization or if instead the patterns observed reflect simple descent from conditions found in their diploid ancestors.

The two *PRE1* homoeologs in *G. hirsutum*,* GhPRE1A* and *GhPRE1D*, share 97% of nucleotide acids in the coding region and 96% amino acid sequence identities. To distinguish between them, we first screened for subgenome‐specific DNA polymorphisms in a series of *Gossypium* accessions. All PRE family genes identified so far, including cotton *PRE1*, have a similar gene architecture with two exons separated by an intron (Fig. 5a). Among the single nucleotide polymorphisms (SNPs) detected, two are located in the second exon (Fig. 5a, underlined). Pyrosequencing of the fragment harboring the two SNPs accurately mirrors the relative amounts of the homoeologous copies in an artificial mixture of *GhPRE1A* and *GhPRE1D* (Fig. S1a). Subsequent analysis of several allotetraploids, including the cultivated *G. hirsutum* and *G. barbadense*, as well as the wild *G. darwinii* and *G. mustelinum*, revealed a striking bias favoring A‐subgenome *PRE1* expression in the ovule and fiber: although the *PRE1A* transcripts were readily detected, the Dt counterparts were undetectable (Figs 5b–d, S1b). However, in *G. tomentosum*, a wild allotetraploid cotton which produces inferior fibers (Fig. S5), this bias is not evident, or even is reversed, with higher expression of the Dt homoeolog (Fig. 5e). We note also that fibers from *G. darwinii* and *G. mustelinum* also are relatively short, much like those of *G. tomentosum*, yet as mentioned *PRE1* is highly biased toward the At homoeolog in these species. These observations collectively suggest that high *PRE1* expression may be necessary but not sufficient for generating highly elongated fiber cells. These expression variations prompted us to further scan polymorphisms in the *PRE1* gene, including the flanking sequences.

### TATA‐box deletion is behind the biased *PRE1* expression

It is well known that *cis* regulatory elements are critical to gene expression and gene expression evolution (Wittkopp & Kalay, 2012). In this light, the strongly biased expression of *PRE1* in allotetraploid cottons suggests a possible presence of intersubgenomic polymorphisms in *cis‐*acting elements that confer the At *PRE1* expression bias, or inactivate the Dt homoeolog (with the exception of *G. tomentosum*).

**Figure 6:**
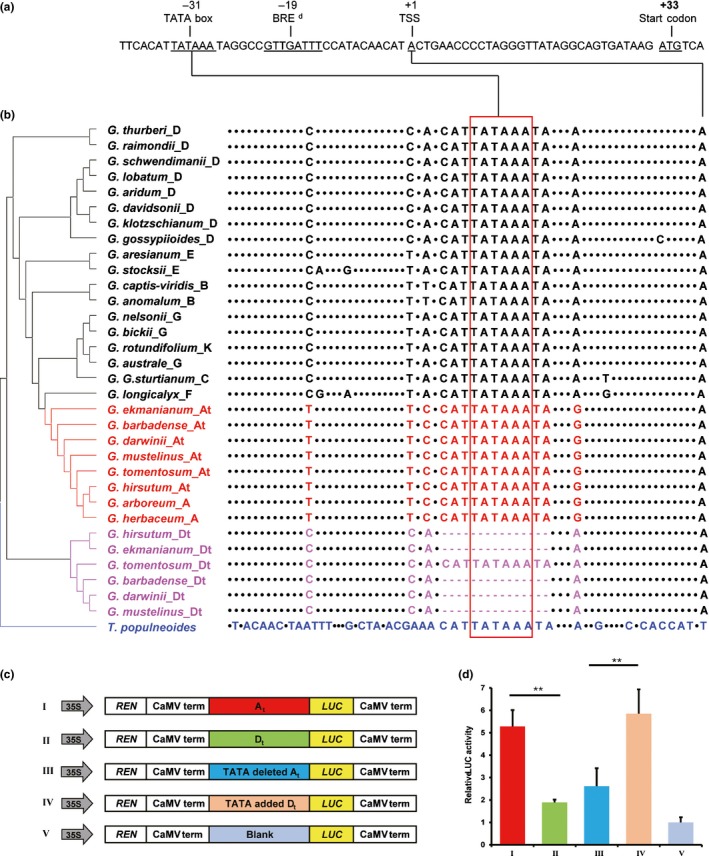
TATA‐box mediates *PACLOBUTRAZOL RESISTANCE 1* (*PRE1*) promoter activity. (a) Sequence of *Gossypium hirsutum GhPRE1A* core promoter fragment. TSS, transcriptional start site. BRE ^d^, downstream transcription factor IIB‐recognition element. (b) *PRE1* core promoter fragments of 32 cotton and the outgroup (*Thespesia populnea*), as in Fig. 4. Promoter sequences of 33 cotton *PRE1* genes were aligned; dashes indicate deletions and dots represent identical nucleotides. The 11‐bp TATA‐box was absent in Dt homoeologous *PRE1* promoters of five allotetraploid species but present in *G. tomentosum*. (c) Schematic map of *GhPRE1A* (At) and *GhPRE1D* (Dt) promoter‐luciferase reporters, in which the TATA‐box fragment was intact, deleted or added. (d) Activities of the promoters in (c), determined by transient expression of a luciferase reporter in tobacco leaves; the value of blank (bottom in c) was set to 1. Data were analyzed by one‐way ANOVA compared to the *GhPRE1A* promoter (At): **, *P *≤* *0.01. Error bars represent SD (*n *=* *3).

In order to map the sequence variations, we performed comparisons of a *c*. 3‐kb genomic fragment spanning the *PRE1* locus (Fig. S6; Table S3). We found that one variation perfectly correlates with *PRE1* expression; in the five allotetraploids with *PRE1A*‐biased expression, the silent D‐subgenome *PRE1D* invariably has an 11‐bp deletion in the promoter region; in *G. tomentosum*, however, which does not show *PRE1A* bias, both homoeologs are intact in this region (Fig. 6a,b). Strikingly, this 11‐bp fragment contains a canonical TATA‐box (5′‐TATAAA‐3′) (Patikoglou *et al*., 1999), which is located 31 nucleotides (nt) upstream to the transcriptional start site (TSS) determined by rapid‐amplification of cDNA (Fig. 6a,b); we term this fragment PRE1‐TATA. Furthermore, an analog of the transcription factor IIB‐recognition element (BRE^d^) (Deng & Roberts, 2007) is present four nucleotides downstream of PRE1‐TATA (Fig. 6a), which further supports it as a genuine TATA‐box. We further noted that all other diploid *Gossypium* species studied, as well as the phylogenetic outgroup *Thespesia populnea*, have an intact PRE1‐TATA (Fig. 6b).

TATA‐box is a core promoter element mediating transcriptional machinery assembling, which in turn directs gene expression (Tirosh *et al*., 2006). Our analysis has revealed that the PRE1‐TATA deletion might be the causal agent for homoeolog‐specific *PRE1* silencing in allotetraploids. To assay the role of PRE1‐TATA in gene expression, we cloned the promoter fragments of *GhPRE1A* and *GhPRE1D*, and used these to drive a firefly LUC reporter gene (Fig. 6c) in tobacco cells and GFP in cotton fiber cells (Fig. S2a), respectively. Consistent with the biased expression pattern in cotton fiber (Fig. 5b–d), the *GhPRE1A* promoter conferred constantly higher reporter activity in tobacco cells than did the *GhPRE1D* promoter (Fig. 6d). Furthermore, deleting the PRE1‐TATA from the *GhPRE1A* promoter was sufficient to reduce reporter activity substantially, and conversely, adding the TATA back to the *GhPRE1D* promoter restored its activity to the level of *GhPRE1A* (Fig. 6d). Consistently, activity of the *GhPRE1A* promoter in cotton fiber cells was greatly reduced when the PRE1‐TATA was removed (Fig. S2b,c). Clearly, PRE1‐TATA is indispensable for high *PRE1* promoter activity.

**Figure 7:**
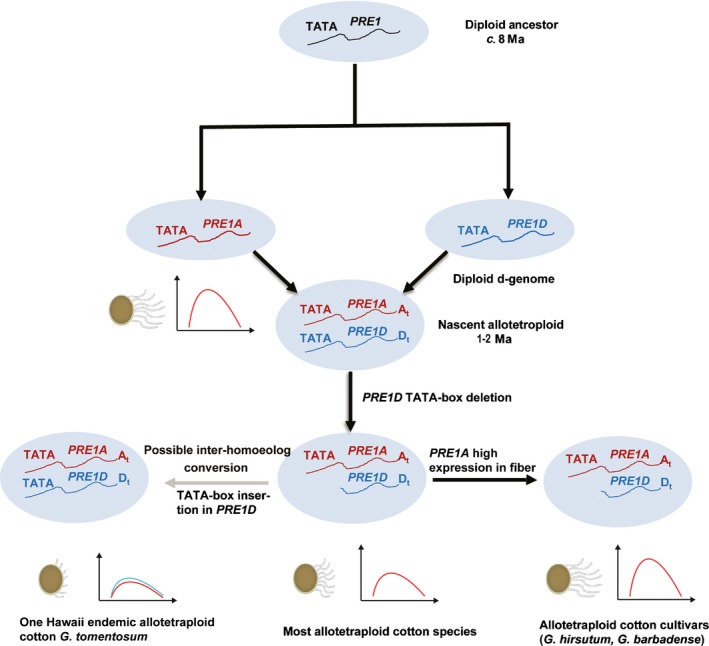
A schematic representation of the TATA‐box indel in D‐subgenome *PACLOBUTRAZOL RESISTANCE 1* (*PRE1*) of allotetraploid cotton species and the association of *PRE1A* expression with spinnable fiber formation. In the cotton genus (*Gossypium*), the *PRE1* gene in all diploid species studied has a canonical TATA‐box in the core promoter. Shortly after allotetraploid (AtDt) formation *c*. 1–2 Myr ago (Ma), a 11‐bp fragment containing the TATA‐box (TATA) of the Dt homoeologous *PRE1* (*PRE1D*) was deleted in the nascent allotetraploid, which was transmitted to its descendants, including most present day wild allotetraploid cotton species. In *G. tomentosum*, however, the TATA‐box was reinserted, possibly through intersubgenomic conversion from the At homoeolog. High expression of *PRE1A* in diploid and allopolyploid cottons is associated with long spinnable fibers. The drawings below the A genome and the polyploid groups represent cotton fibers (left) and expression of *PRE1* homoeologs (right).

Modifications of *cis*‐elements often lead to expression changes in spatial patterns or transcription levels of genes, and this is a key mechanism of adaptive evolution (Wray, 2007; Carroll, 2008; Grover *et al*., 2012; Wittkopp & Kalay, 2012; Lemmon *et al*., 2014). To examine the correlation between the *PRE1* TATA‐box variation and cotton fiber phenotypes, we surveyed 163 accessions of diploid and allotetraploid cottons including wild species, semi‐wild and domesticated races of distinct geographical origins (Tables S1, S4). The canonical TATA‐box element is present in the *PRE1* promoter of all 20 representative diploid cotton species of different genome types surveyed (Fig. 6b). However, as noted above, PRE1‐TATA is deleted from *PRE1D* of five of the six allopolyploid species studied, the exception being *G. tomentosum* in which it is present (Figs 5e, 7).

Thus, *G. tomentosum* lacks the expression bias (Fig. 5e) and also the *PRE1D* TATA deletion (Fig. 6b). These two observations are not independent, as the TATA‐box is indispensable for *PRE1* promoter activity (Fig. 6c,d). It may be that, in this species, for reasons that are unclear, the two homoeologs are under strong *trans*‐regulation, thereby upregulating both the At and Dt *PRE1* genes. From a phylogenetic perspective, accumulated evidence indicates that this Hawaiian Islands endemic is not basal within the polyploid clade, but that it is instead nested within the polyploids in a position close to the *G. hirsutum* clade (Grover *et al*., 2012, 2015). Thus, the manner in which *G. tomentosum* acquired an intact as opposed to deleted TATA element in its Dt homoeolog is of interest. One possibility is that it reflects simple inheritance from the D‐genome diploid ancestor, but this would require independent loss of the TATA element in at least two other allopolyploid lineages and at identical positions; thus we view this scenario as unlikely. A second alternative related to this first idea is that of vertical inheritance from the D‐genome diploid ancestor but that the true phylogeny differs from that presently indicated by a wealth of data (Grover *et al*., 2012, 2015); we view this scenario as equally unlikely. A final possibility is that of ‘gene conversion’; that is, nonreciprocal homoeologous exchange involving physical contact of the two homoeologs, such that the indel was ‘corrected’ by the *PRE1A* copy (Fig. 7). Interhomoeolog exchange has been described in cotton previously (Salmon *et al*., 2010; Flagel *et al*., 2012; Guo *et al*., 2014), and we note other evidence for this process affecting *PRE1* genes elsewhere in this paper (see discussion of *G. ekmanianum*, in the results). If, indeed, *G. tomentosum* acquired its *PRE1D* TATA element via gene conversion, the converted region was quite small, as it remains flanked by diagnostic D‐genome‐specific SNPs (Fig. 6b).

## Discussion

PACLOBUTRAZOL RESISTANCE (PRE) proteins in plants function via protein–protein interactions and act as a hub integrating multiple signaling pathways to regulate cell growth and development (Lee *et al*., 2006; Wang *et al*., 2009; Zhang *et al*., 2009; Schlereth *et al*., 2010). In addition, *PRE* genes themselves are also regulated by phytohormones, such as gibberellic acid (GA) (Park *et al*., 2013). Our previous investigation showed that the homeodomain leucine zipper (HD‐ZIP) IV transcription factor *Gossypium hirsutum* (*Gh*) GhHOX3 is a core regulator of cotton fiber elongation, it interacts with other HD‐ZIP IV factors, leading to enhanced transcriptional activation of downstream genes, including *GhRDL1* and *GhEXPA1*, which promote cell wall loosening (Wang *et al*., 2004; Xu *et al*., 2013). The GA signaling repressor DELLA interferes with this process through competitive binding to GhHOX3, thus transducing GA signal to cotton fiber growth (Shan *et al*., 2014). Similar interaction also functions in Arabidopsis in mediating hypocotyl epidermal cell growth (Rombola‐Caldentey *et al*., 2014), suggesting that this is a general mechanism that promotes plant cell growth.

In the present investigation, we identified *GhPRE1A* from the *GhHOX3* co‐suppression lines and its expression was repressed following *GhHOX3* silencing. However, the *GhPRE1* promoter does not contain an L1 *cis*‐element recognized by HD‐ZIP IV factors, and thus the repression may be an indirect effect of GhHOX3. Similar to the positive role of reported PREs in plant cell growth (Hyun & Lee, 2006; Lee *et al*., 2006), GhPRE1 acts as a positive regulator of cotton fiber elongation, but whether its function involves the GhHOX3‐DELLA complex is not clear at this time. One possibility is that it acts in bridging hormone (such as GA and brassinosteroid (BR)) signaling and the cell autonomous pathways to control fiber cell growth.

Phenotypic novelty in evolution, resulting from either natural or under strong directional human selection, can target coding regions or variation in regulatory elements (Wray, 2007; Carroll, 2008; Wittkopp & Kalay, 2012; Lemmon *et al*., 2014). One example is of an insertion of a TATA‐box in the promoter of an Iron‐Regulated Transporter1 (IRT1) gene in apples, leading to enhanced tolerance to iron (Zhang *et al*., 2016). A second relevant example involves tomatoes (*Solanum lycopersicum*), which are cross‐pollinated with long exerted stigmas in the wild but have included stigmas in cultivated forms (Rick *et al*., 1977; Chen *et al*., 2007). Stigma length is controlled by *Style2.1*, which encodes a PRE‐like protein, and it is the novel presence of two indels in the promoter that reduced *Style2.1* expression in domesticated tomatoes (Chen *et al*., 2007).

Cotton *PRE1* also varies in its promoter sequence, with a fragment deletion directly removing the core regulatory element, the TATA‐box. In allotetraploid cottons, deletion of the TATA‐box in the D‐subgenome *PRE1* silenced its expression, resulting in A‐subgenome‐specific expression. Notably, in *G. raimondii*, the closest D‐genome progenitor species with short fibers, *PRE1* expression levels are low in ovules, whereas *PRE1* is highly expressed in fiber cells in the allopolyploids and the diploid A‐genome cottons. Thus, high *PRE1* expression in fiber is a characteristic both of some diploids and of all allopolyploids. It seems likely from the evidence presented here that at the time of initial polyploidization, the highly expressed *PRE1A* inherited from the A‐genome parent may have helped stimulate fiber elongation in nascent allotetraploid cottons, which thereby acquired an expression bias toward the parental A‐subgenome *PRE1*, whereas the Dt homoeolog was further silenced as a result of the TATA‐box deletion from its promoter (Fig. 7). The exception, observed in *G. tomentosum,* is most likely due to interhomoeolog gene conversion from the A‐ to D‐ *PRE1* homoeolog after diversification of the primary allotetraploid cotton lineages. Subsequently, and in parallel in the two‐cultivated species *G. barbadense* and *G. hirsutum*, expression levels became enhanced as a consequence of the domestication process (Fig. 7). Probably not coincidently, variations in the *PRE* gene regulatory elements are closely related to key agronomic traits in both tomato and cotton, which suggests a recurring role of *PRE* gene expression patterns in crop evolution.

The widely cultivated upland cotton *G. hirsutum* and the high‐quality extra‐long staple (ELS) cotton *G. barbadense* have experienced a relatively long history of evolution following the merger of two sets of genomes. Transcriptome sequencing has repeatedly demonstrated that a high fraction (often 20–40%) of genes in *G. hirsutum* exhibit A‐ or D‐subgenome biased expression (Yoo & Wendel, 2014; Liu *et al*., 2015; Zhang *et al*., 2015). In particular, in *G. hirsutum* the myeloblastosis (MYB)‐MIXTA‐like transcription factor gene *GhMML4* (*GhMML4_D12*), a key regulator of lint (spinnable fiber) development, shows a strong subgenome‐biased expression (Wu *et al*., 2018). We speculate that that selection has shaped gene expression variation through its action on underlying variation in multiple regulatory elements throughout the genome. The fact that the two progenitor diploid genomes vary two‐fold in size and that they evolved in isolation on different continents for 5–10 Myr, suggests that *cis*‐element divergence at the diploid level would have been prevalent at the time of initial polyploid formation. With the mounting genome sequences and tools available, extensive comparisons of sequence variations in relation to gene expression patterns will undoubtedly uncover additional examples of the type of evolution and selection described here.

We note that higher expression of *PRE1A* appears to have evolved in the diploid A‐genome species, in association with the first appearance of long, spinnable fibers (Fig. 3c). Yet this higher expression did not depend solely on the TATA‐box, because this is universally present among the diploid orthologs, including A and D. After AD allotetraploid formation, the TATA deletion from the Dt (tetraploid) homoeolog generated biased expression, raising the question about the functional relevance of the TATA deletion. In this respect we envision two possible scenarios, one neutral and one driven by differential homoeolog selection. Under the neutral scenario, the deletion was not functionally relevant; the *PRE1D* TATA deletion was inconsequential, perhaps because of the redundancy offered by its *PRE1A* homoeolog. If instead natural selection favored the *PRE1A* homoeolog, the deletion may have arisen following selective fixation of a novel TATA deletion in the *PRE1D* copy, concomitant with or followed by both natural and human‐mediated selection for enhanced *PRE1A* expression in fibers. As the atypical basic helix‐loop‐helixes exert biological functions in partnership with other transcription factors, finding the GhPRE1 interacting partners holds the key to understand the role of PRE1A in the regulatory network of cotton fiber elongation, which will further help us understand the association of *PRE1A* high expression with long fiber formation.

Interestingly, overexpression of *GhPRE1A* in cotton not only promoted fiber elongation, but also improved fiber strength (Table 1). A plausible explanation is that formation of the fiber strength trait involves coordinated cell wall biosynthesis and extension. Given these findings, it will be of interest to explore the relationship between PRE1 expression and fiber length/ strength in other *Gossypium* species and varieties, particularly inasmuch as there exists considerable infraspecific varietal variation and interspecific differences in fiber length.

## Author contributions

B.Z., J.F.W. and X‐Y.C. conceived the research; B.Z., J‐F.C. and Z‐W.C. performed the experiments; B.Z., G‐J.H, J.F.W. L‐Y.W., X‐X.S., Y‐B.M., L‐J.W. and T‐Z.Z. contributed materials and/or analyzed data; X‐Y.C., J.F.W. B.Z., J‐F.C. and G‐J.H. wrote the article.

## Supporting information

Please note: Wiley Blackwell are not responsible for the content or functionality of any Supporting Information supplied by the authors. Any queries (other than missing material) should be directed to the *New Phytologist* Central Office.

**Fig. S1 **Pyrosequencing of homoeologous *PRE1* transcripts of allotetraploid cottons.
**Fig. S2 **TATA‐box mediates *PRE1* promoter activity in cotton fiber cells.
**Fig. S3** *GhPRE1A* promotes cell elongation in *Arabidopsis*.
**Fig. S4** *GhPRE1* is expressed in fibers during the fast elongation period in fiber‐producing cotton species.
**Fig. S5 **View of fibers (seed trichomes) of five allotetraploid cotton species.
**Fig. S6 **The *PRE1* locus in the genomes of four cotton species.
**Table S3 **The *PRE1* loci in reported cotton species

**Table S1 **Diploid and allotetraploid cottons surveyed in this investigation
**Table S2 **Primers used in this investigation
**Table S4 **Distribution of the TATA‐box indel in cotton accessions
