# Supplementary material for: Core cis‐element variation confers subgenome‐biased expression of a transcription factor that functions in cotton fiber elongation
Source: New Phytol. 2018 Feb 21;218(3):1061–75. doi: 10.1111/nph.15063 (PMC6079642; doi:10.1111/nph.15063)
Supplement: Supplementary file 1 — Fig. S1 Pyrosequencing of homoeologous PRE1 transcripts of allotetraploid cottons. Fig. S2 TATA‐box mediates PRE1 promoter activity in cotton fiber cells. Fig. S3 GhPRE1A promotes cell elongation in Arabidopsis. Fig. S4 GhPRE1 is expressed in fibers during the fast elongation period in fiber‐producing cotton species. Fig. S5 View of fibers (seed trichomes) of five allotetraploid cotton species. Fig. S6 The PRE1 locus in the genomes of four cotton species. Table S3 The PRE1 loci in reported cotton species [file NPH-218-1061-s001.pdf]

New Phytologist Supporting Information: Figs S1-S6 & Table S3

Article title: Core cis-element variation confers subgenome-biased expression of a transcription factor that functions in cotton fiber elongation

Authors: Bo Zhao, Jun-Feng Cao, Guan-Jing Hu, Zhi-Wen Chen, Lu-Yao Wang, Xiao-Xia Shangguan, Ling-Jian Wang, Ying-Bo Mao, Tian-Zhen Zhang, Jonathan F. Wendel and Xiao-Ya Chen

Article acceptance date: 17 January 2018

The following Supporting Information is available for this article:

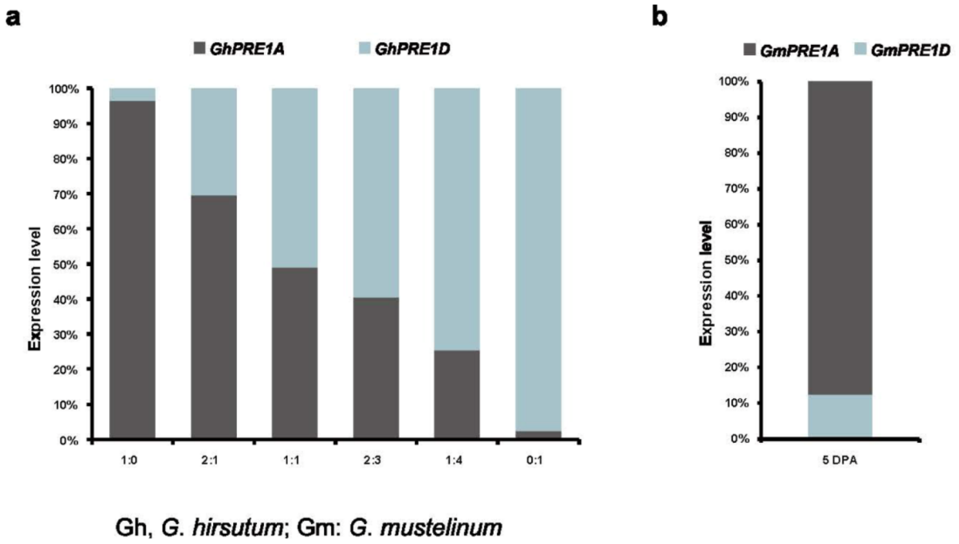

Fig. S1 Pyrosequencing of homoeologous *PRE1* transcripts of allotetraploid cottons.

a, Determination of relative transcript abundance of *GhPRE1A* and *GhPRE1D* in artificial mixtures of the transcripts at six different ratios. **b**, Relative expression levels of *PRE1* homoeologs (*GmPRE1A* and *GmPRE1D*) in the 5-DPA fiber cells of *G. mustelinum*.

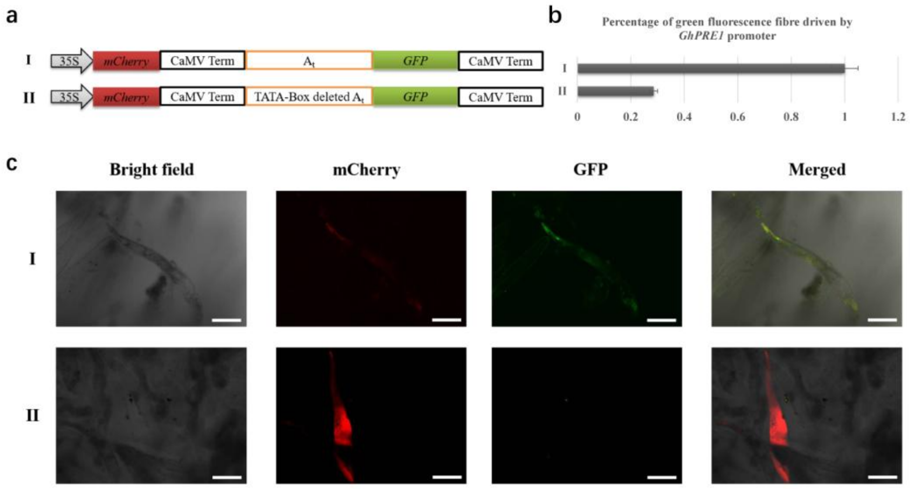

Fig. S2 TATA-box mediates *PRE1* promoter activity in cotton fiber cells. **a**, Schematic map of *GhPRE1A* promoter-green fluorescence protein reporters, the TATA-box fragment was intact (At, above) or deleted (TATA-del-At, below). **b**, Percentage of green fluorescence fiber driven by *GhPRE1*At or TATA-deleted-At promoter, respectively. Six ovules were observed with 95.67% green fluorescence fiber in wild-type (I) and 28.57% in TATA-deleted (II) *GhPRE1At* promoter, respectively. error bars indicate SD (*n*=3). **c**, Activities of the wild-type (I) and the TATA-deleted (II) *GhPER1At* promoters determined by transient expression of a green fluorescence protein reporter in cotton fiber cells. The 1-DPA ovules were bombarded and the GFP signal was examined 1 day later. Bar 100 µm.

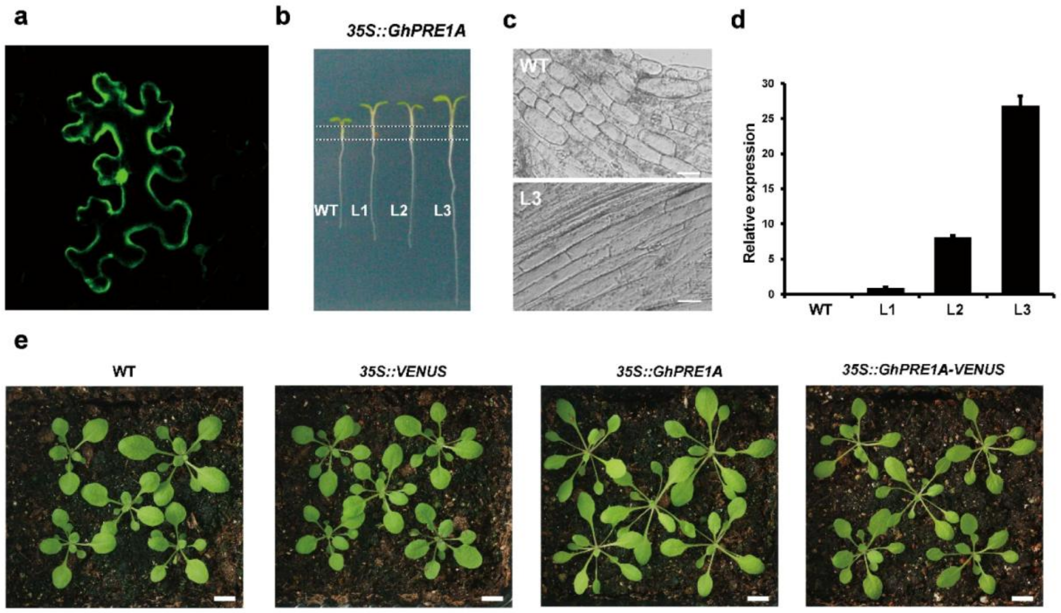

Fig. S3 *GhPRE1A* promotes cell elongation in *Arabidopsis*.

a, Subcellular localization of GhPRE1A-VENUS in tobacco leaf cells. **b-e**, Phenotypes of transgenic *A. thaliana* overexpressing *GhPRE1A* (*35S::GhPRE1A*). **b**, The root is longer in transgenic lines, L1-L3 are transgenic lines. **c**, *GhPRE1A* expression increased length of hypocotyl cells, as observed under an optical microscope, bar = 100 μm. **d**, Expression of *GhPRE1A* in transgenic *Arabidopsis* lines, transcripts of the 7-day old plants were analyzed by qRT-PCR; error bars indicate SD (*n*=3). **e**, View of *Arabidopsis* plants (14-day old), the petiole was longer when *GhPRE1A* was overexpressed. Bar = 1cm.

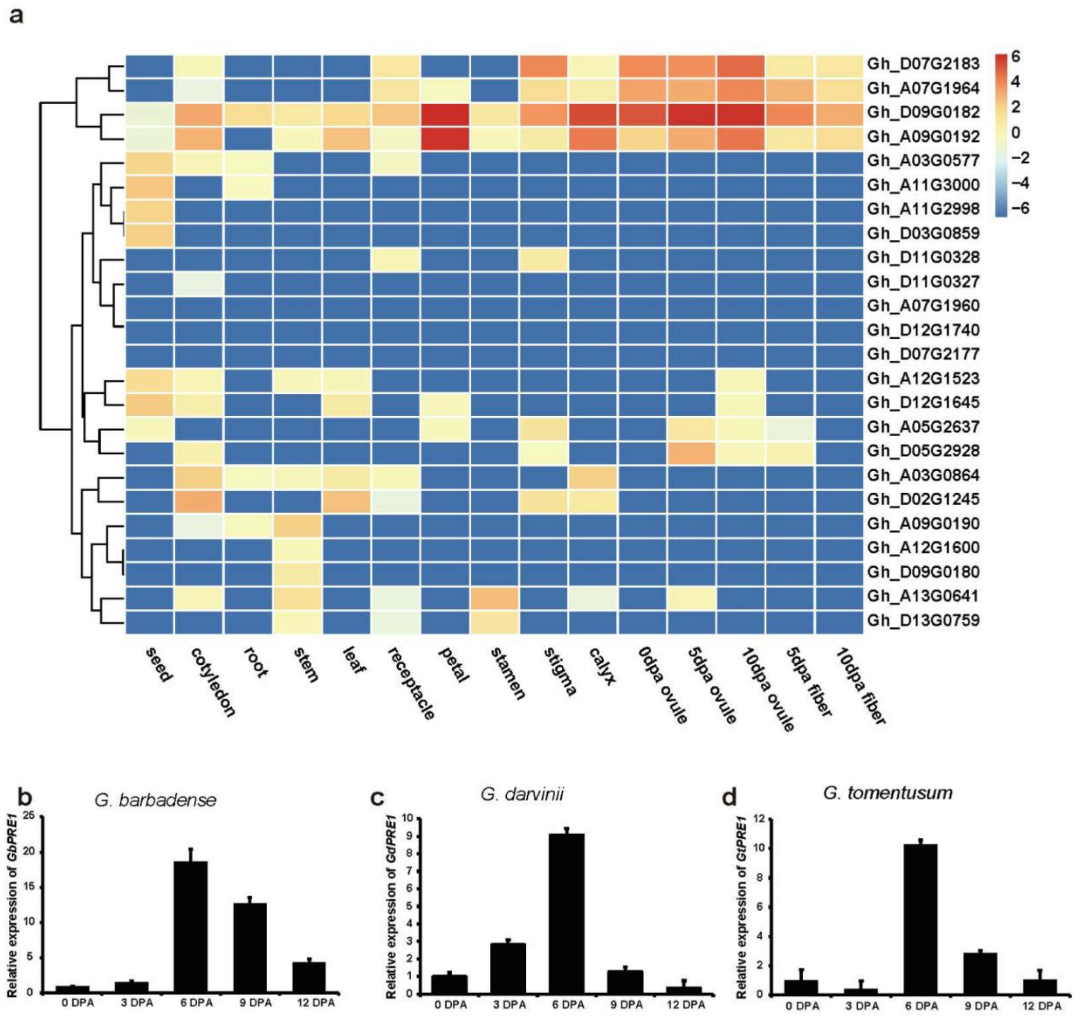

Fig. S4 *GhPRE1* is expressed in fiber at fast elongation period in fiber-producing cotton species.

a, The heatmap showing expression of *PRE* family genes in *G. hirsutum*. The genes shown are *GhPRE4s* (Gh_D07G2183 and Gh_A07G1964), *GhPRE5s* (Gh_D09G0182 and Gh_A09G0192) See also legend to Figure 3a. **b-d**, Expression of *PRE1* orthologs in allotetraploid fiber-producing cottons including *G. barbadense* (**b**), *G. darwinii* (**c**), *G. tomentosum* (**d**), analyzed by qRT-PCR; error bars indicate SD (*n*=3).

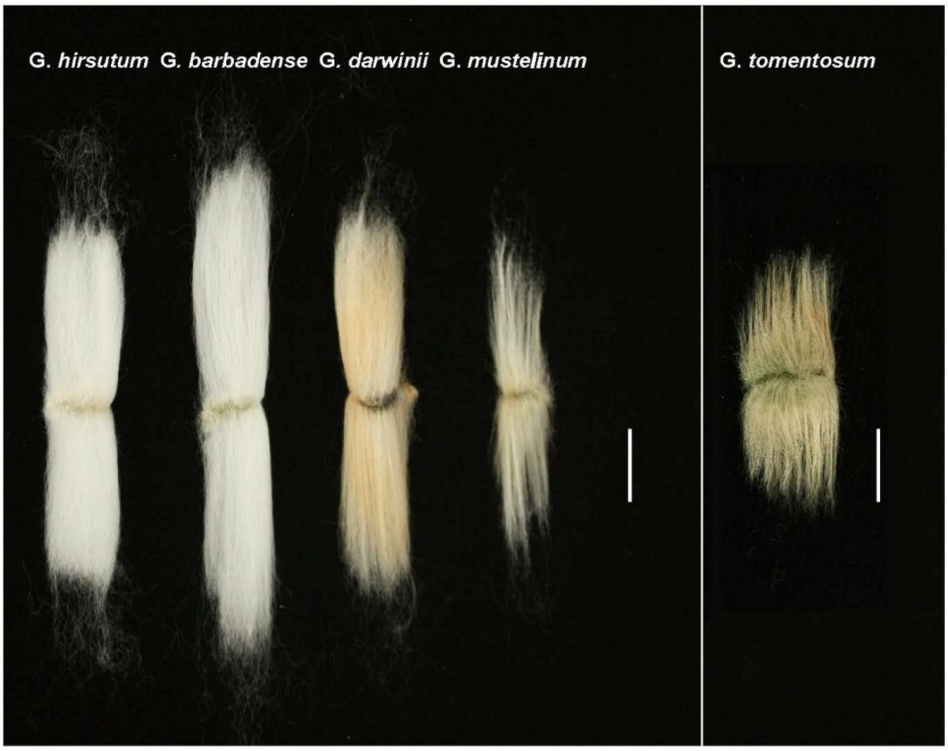

Fig. S5 View of fiber (seed trichomes) of five allotetraploid cotton species. Bar = 1cm.

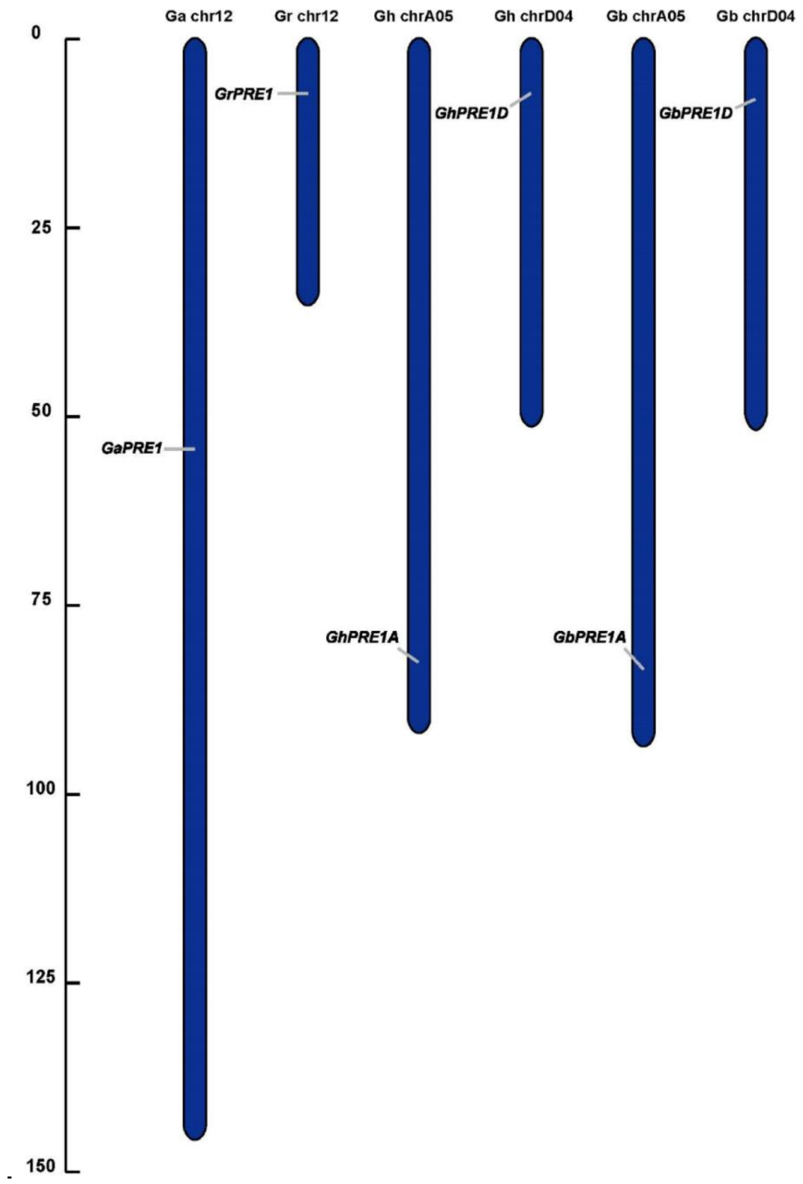

Fig. S6 The *PRE1* locus in the genomes of four cotton species.

The *GhPRE1A* promoter used in this investigation is located on chromosome 5, 82536086-82639085 (see ref (Zhang et al., 2015)). Promoters of similar length of other *PRE1* genes were used for comparative analyses. Ga, *G. arboreum*; Gb, *G. barbadense*; Gh, *G. hirsutum*, Gr, *G. raimondii*.

Table S3 The *PRE1* loci in reported cotton species

Gene	Chromosome	Start	End	Promoter region analyzed
GrPRE1	Chr12	7361834	7362402	7358834--7361833
GhPRE1D	D04	7304311	7304656	7301311--7304311
GhPRE1A	A05	82636434	82636085 (-)	82636086--82639085
GbPRE1D	D04	8080099	8080448	8077099--8080098
GbPRE1A	A05	83573117	83575075 (-)	83575076--83578075
GaPRE1	Chr12	54434101	54434450 (-)	54434451--54437450

(-): Position in the antisense strand. Ga, *G. arboreum*; Gb, *G. barbadense*; Gh, *G. hirsutum*, Gr, *G. raimondii*.

References

Zhang T, Hu Y, Jiang W, Fang L, Guan X, Chen J, Zhang J, Saski CA, Scheffler BE, Stelly DM, et al. 2015. Sequencing of allotetraploid cotton (*Gossypium hirsutum* L. acc. TM-1) provides a resource for fiber improvement. *Nature Biotechnology* **33**: 531-537.
